# Ten simple rules for designing graphical abstracts

**DOI:** 10.1371/journal.pcbi.1011789

**Published:** 2024-02-01

**Authors:** Helena Klara Jambor, Martin Bornhäuser

**Affiliations:** 1 National Center for Tumor Diseases—University Cancer Center (NCT-UCC), Universitätsklinikum Carl Gustav Carus an der Technischen Universität Dresden, Germany; 2 Medical Clinic 1, Universitätsklinikum Carl Gustav Carus an der Technischen Universität Dresden, Germany; Dassault Systemes BIOVIA, UNITED STATES

## Introduction

Explanatory graphics that summarize knowledge are common in science communication. These graphics integrate new insights with the existing body of knowledge in a particular field of research. Explanatory graphics have been widely used in chemistry for many years to depict structures [1], and they have now gained popularity across various scientific disciplines as graphical abstracts [2]. Scientific journals are increasingly asking authors to provide graphical abstracts along with a paper to attract audiences online and on social media. These graphical abstracts are prominently displayed on the journals websites, embellishing the table of contents, and serving as a visual pendant to the written abstract. Due to this usage, graphical abstracts are also referred to as “TOC” image or “thumbnail views.”

Graphical abstracts are not intended to provide a complete understanding of a research article, even though they are often presented online with just the title of the work. A study confirmed graphical abstracts by themselves are insufficient to comprehend the key message of a paper [3]. Instead these visuals serve to attract attention and are meant to be read in conjunction with the written abstract. According to Cell press guidelines, graphical abstracts should inspire audiences to browse, stimulate their interdisciplinary curiosity, and allow them to rapidly screen for papers in journals [4]. As graphical abstracts are a relatively recent addition to the publishing landscape, quantitative data on their usage and usefulness are still limited. However, early analyses indicate that while graphical abstracts do not necessarily increase full-text reads or citations, they do enhance the abstract views [5] and boost altimetric attention scores of articles [6].

Like other explanatory visualizations, graphical abstracts have common features such as a central visual element, often icons, diagrams or photos, explanatory text, and use clear layout and color schemes to increase readability. These elements are often structured using arrows and lines and enhanced with color. The design elements of graphical abstracts were recently quantified in a research study that classified graphical abstracts based on their overall organization [2]. In their work, Hullman and Bach revealed the diversity of graphical abstracts in the current literature, and in particular, the many possibilities to use layout for readability. They also pinpointed common problems associated with graphical abstracts, such as inconsistent visual styles, unclear relationships between pictures, and missing annotations. These challenges were also identified in a complementary qualitative study of graphical abstracts [7].

Training of scientists, especially early career researchers, in the art of crafting comprehensible and attractive graphical abstract has been somewhat lagging. A brief guide for graphical abstract design is available for medical writers [7] and for creating overview figures [8]. However, most scientist are not trained in data visualizations or visual communication [9,10], and even less so in creating explanatory visuals of their research. It’s important to note that visual design is a nontrivial endeavor. Publishing houses, journals, and major research institutes often employ visual teams to create attractive explanatory figures for scientific data.

**Fig 1:**
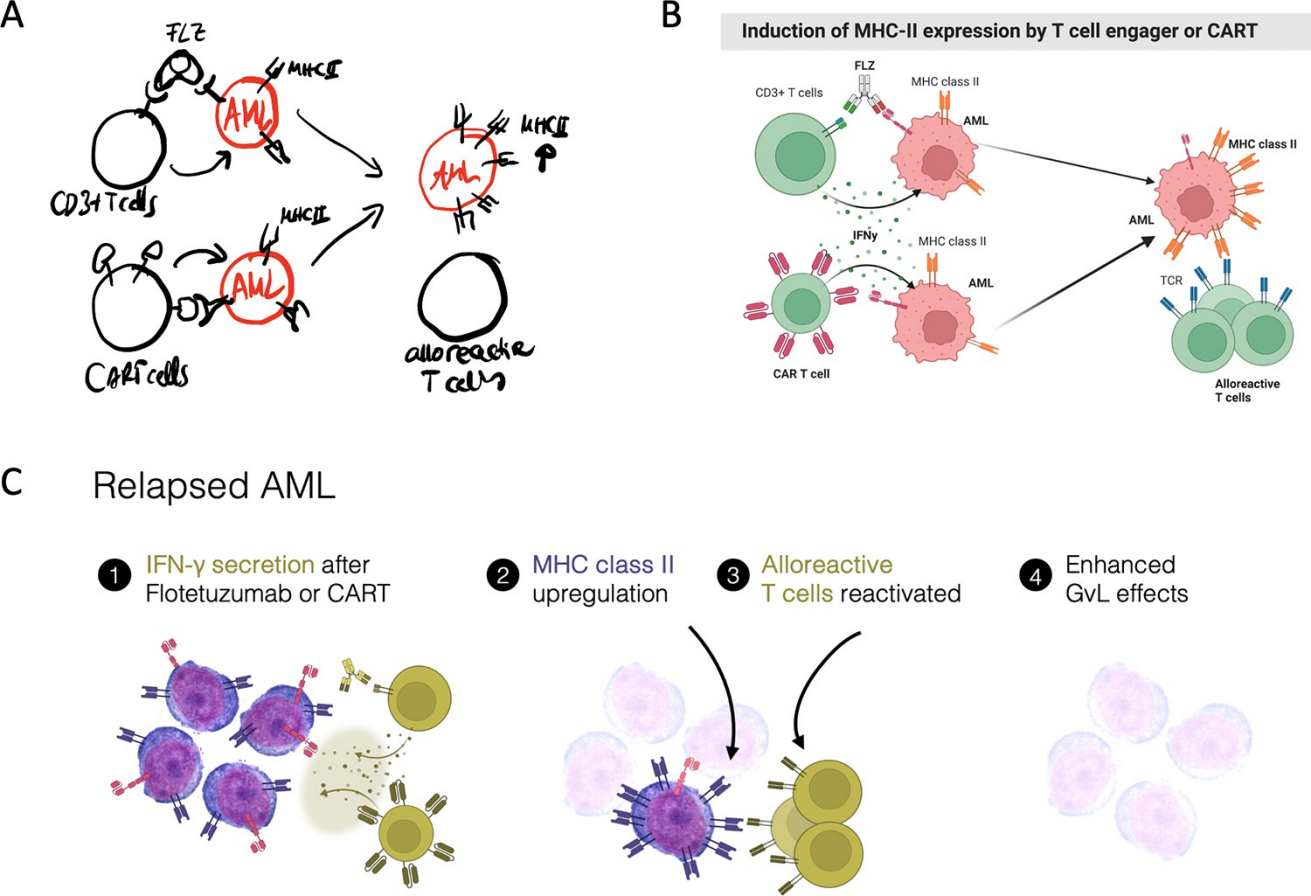
The evolution of a graphical abstract, from sketch (A) to a rapid Biorender draft (B) and final implementation in a graphical software program (C). All drawings by HKJ, licensed under CC0, https://doi.org/10.6084/m9.figshare.24486061.v1.

Here, we present 10 simple rules for designing graphical abstracts. The 10 rules are informed by our experience teaching biologists, clinicians, students, and established scientists, and from jointly preparing graphical abstracts for publications and grants (Fig 1). The article discusses all aspects of graphical abstract preparation, from foundational decisions about the message and the key visuals (1 to 3), to designing the layout (4 to 6), and complementing the design with text (7) and color (8). We also provide an overview of tools and software commonly used for making graphical abstracts (9) and highlight the benefit of feedback in the process (10). The order of the 10 rules reflects our “design pipeline” from starting with a draft to implementing the draft electronically; however, as with all creative processes, you are encouraged to adapt the process to your own style.

## Rule 1: Key message for audience

Before embarking on the design of a graphical abstract, it is essential to know your message. This tip is not specific to graphical abstracts, but also essential for producing an understandable and clear visualization. The process of defining a key message varies. Some start with doodling on a post-it, some with key visuals, and some by iteratively shortening the abstract to 1 or 2 punchy sentences. Ideally already at this stage co-authors are involved and provide feedback (see #10). Recent tools, such as chatGPT, may be helpful in facilitating a dynamic exchange and the concise distillation of the core message. Whichever route is yours, without a clear central message, it will be impossible to design a clear graphical abstract and reach the goal of visually summarizing your research paper.

## Rule 2: Pictures and pictograms

The key components of every graphical abstract are the visual elements. Most often, graphical abstracts include pictograms or symbols and, less commonly, iconic microscope (Fig 1) or photographic images, or data (see #5). Pictograms may also be hand-drawn, but mostly biologists use simple shapes, circles, ellipses, and rectangles, when creating pictograms from scratch. In recent years, numerous icon collections have become available, many of which are free to re-use and do not always need attribution. In most icon repositories pictograms can be downloaded as PNG (Portable Network Graphic), a raster-graphics format for lossless data compression. PNGs are ready to use in graphic software but not adaptable. Alternatively, icons are provided as SVG (Scalable Vector Graphics), an image format that can also be used interactively on the web and is fully adaptable in appearance with graphic software.

For general icons, many repositories exist for simple icons:

- PowerPoint offers inbuild image and icon libraries and many pre-drawn shapes that are free to use.
- Fontawesome (https://fontawesome.com) is a Unicode-based icon library that can be installed locally as a font for graphic programs, downloaded as full icon library, or downloaded as individual SVG images.
- Nounproject (https://thenounproject.com) is a large repository sourcing icons from various designers. Hence, the available icons are vast, but also not matched in style. These icons can be used for free with attribution as SVG or PNG.
- SVGrepo (https://www.svgrepo.com) is the largest SVG icon library, which additionally provides search functions for icon style and appearances such as color, rounded or sharp icons.

Biology and Medicine require specific icons which are available in the following repositories:

- Phylopic (https://www.phylopic.org/) offers shapes of numerous animals, plants, and further model organisms, e.g., for phylogenetic trees.
- The EBI (https://www.ebi.ac.uk/style-lab/general/fonts/v1.3/) provides some general scientific icons.
- Reactome (https://reactome.org/icon-lib) provides scientific pictograms and chemical drawings for free re-use and encourages the upload of user-designed pictograms for sharing with the scientific community.
- Smart (https://smart.servier.com/) is a free collection of medical drawings from Servier Medical Art and can be downloaded as a full slide-deck and used with attribution.
- Bioicons (https://bioicons.com/) is an expanding set of biology and laboratory icons from Petri dishes to model organisms available under free licenses (CC0). Initially by Servier, the drawings are expanded with user provided samples.
- Health Icons (https://healthicons.org/) is a global volunteer effort to create common icons for many specialized medical scenarios available under creative commons license (CC0).

**Fig 2:**
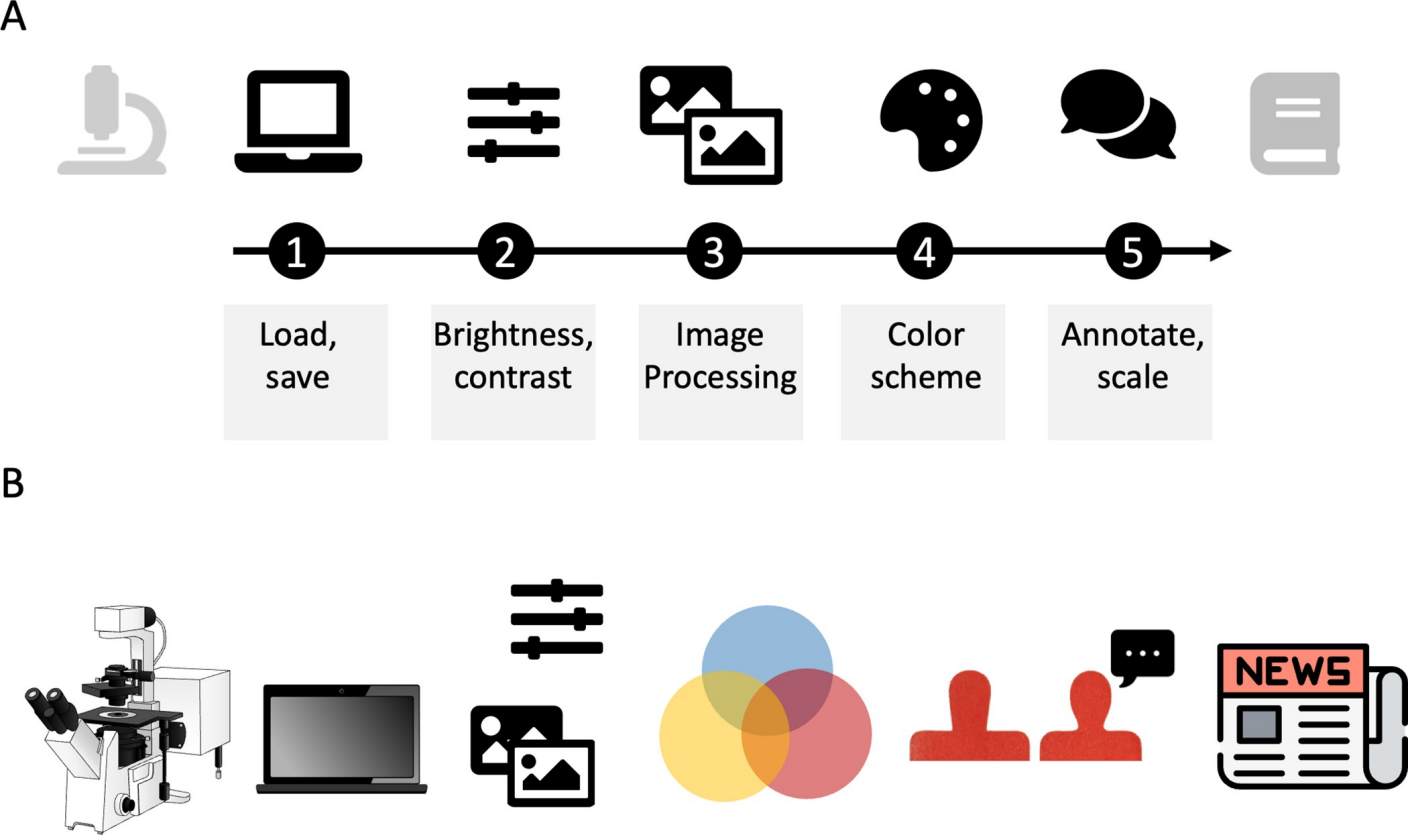
(A) All pictograms used have similar overall appearance (color, size, design, modified from [11]). (B) Poor combination of pictogram for the same workflow: pictograms have different overall appearance. Icons in A: Fontawesome, Fonticons, Inc. Icons in B: Microscope: Bioicons DBCLS https://togotv.dbcls.jp/en/pics.html is licensed under CC-BY 4.0; Laptop: Icon by Simon Dürr https://twitter.com/simonduerr is licensed under CC0 https://creativecommons.org/publicdomain/zero/1.0/; Image/slider: see A; Colors and people: drawn by HKJ; Newspaper: https://www.svgrepo.com/svg/301104/newspaper-news, CC0.

In a graphical abstract, all icons should have a similar overall appearance, meaning the same line-width, color scheme, and level of detail. Icons from the same source and/or the same designer usually have such a similar look. Fig 2A and 2B shows 2 versions of a graphical abstract with a poor and improved icon combination. If icons from several sources are combined, you could match their style by adapting the SVG-pictograms in graphic software.

For inspiration, you may wish to explore one of the earliest icon libraries, the ISOTYPE. The ISOTYPE system was developed by Otto Neurath in the 1920ies in Vienna as a visual communication tool for low-literate populations. The designs are from Gerd Arntz and were later continued by Marie Neurath (http://gerdarntz.org/isotype.html).

## Rule 3: Data and charts as key visual

**Fig 3:**
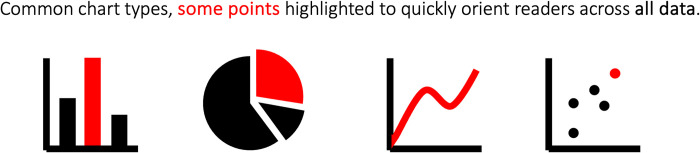
Common chart types with few data categories/points each that convey high level information. Note that the core message (increases, is most, one third…) is communicated without axis details, labels, and legends.

At times pictograms cannot sufficiently represent a key message. You then may wish to include data or charts in your graphical abstracts. When your data are medical, microscopy, or photo images they may be self-explanatory in graphical abstracts. When you want to instead include data plots, you should aim for chart types that are understandable even in the short view time of graphical abstracts. Most of us are familiar with bar charts, which are the most common chart type in scientific publications [12,13], and with pie and line charts, plot types we usually learn in school (Fig 3). These charts employ core principles of visual perception: in bar charts we almost intuitively compare lengths, in pie charts the slice areas, and in line charts we look for up- or downward trends [14].

When it is necessary to signify the use of a specific method in graphical abstracts, sometimes method-specific charts are employed as visual placeholders. For example, t-SNE plots may represent single-cell data, red/green heatmaps can denote microarray data, and circoid plots are indicative of genomic approaches. However, it’s important to note that readers of your graphical abstract are unlikely to delve into the details in these advanced graphics. In such instances, a simple version of that charts should be used, featuring only a handful of data points or categories. Details like tick marks, axis label, and legends can be omitted. For a comprehensive understanding of different chart types and their appropriate use a valuable resources is the Data Visualization Catalogue (https://datavizcatalogue.com/).

## Rule 4: Layout: The dimensions

**Fig 4:**
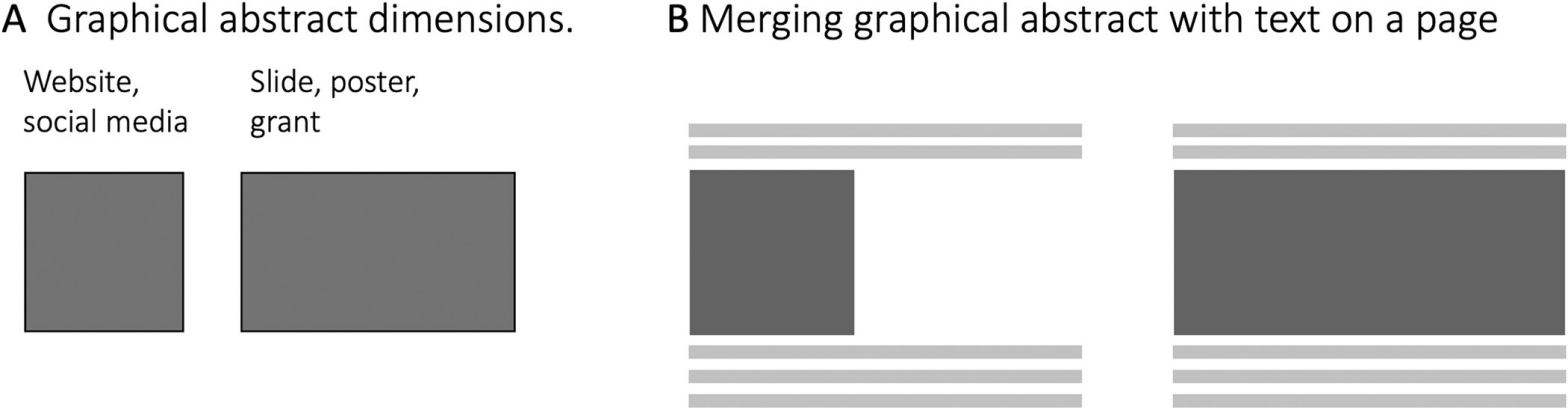
Different dimensions (A) and how they merge with text on a page (B).

Layout describes the organization of visual elements on the page (Fig 4). First, consider the space that you have available to fill. A graphical abstract for a journal website is typically shown as a square and rarely in rectangle format (Fig 4A). On many websites and applications, the graphical abstract has a final size not much larger than a postage stamp. When a graphical abstract is the first figure of an article, poster, or grant application, you may also opt for a landscape rectangle format. Whenever choosing a layout, you should consider how to fill the area best. In grant applications space is very limited, filling the entire width of a line may then be a best choice to not waste precious space (Fig 4B).

## Rule 5: Layout: Reading direction

The layout should provide a clear entry point into your graphical abstract. Typically, we read from left to right, and top to bottom in either columns or rows. You should therefore arrange all elements of the graphical abstract along your chosen reading direction [15].

**Fig 5:**
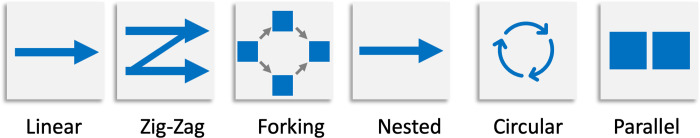
Different layouts for graphical abstracts with clear start and end.

For depiction of linear processes that have a clear beginning and end, an organization from left to right is most suitable: time is usually shown as the independent variable on the x-axis in graphs. Linear processes are workflows, experimental pipelines, embryonic development, cellular differentiation, or disease progression. Alternatively, you can consider a circular layout for cyclic events such as daily or annual events, metabolic cycles, or processes like cell division. For static observations, e.g., contrasting 2 scenarios or providing 2 levels of details for 1 scenario, you could consider 2 parallel or nested organization [2]. Fig 5 summarizes the most common organizational layouts of graphical abstracts.

## Rule 6: Connecting the elements: Arrows and arrangement

Arrows are a key element for all explanatory graphs and visual abstracts. With arrows, we connect text, pictograms, images, and charts into a sequential narrative or “storyline” and consequently they are the most common graphical element in explanatory life science figures [16]. Arrows can reinforce your chosen reading direction but arrows can also signal any exception from this reading direction. A clear layout supported by arrows helps to quickly orient your audiences in a visualization.

**Fig 6:**
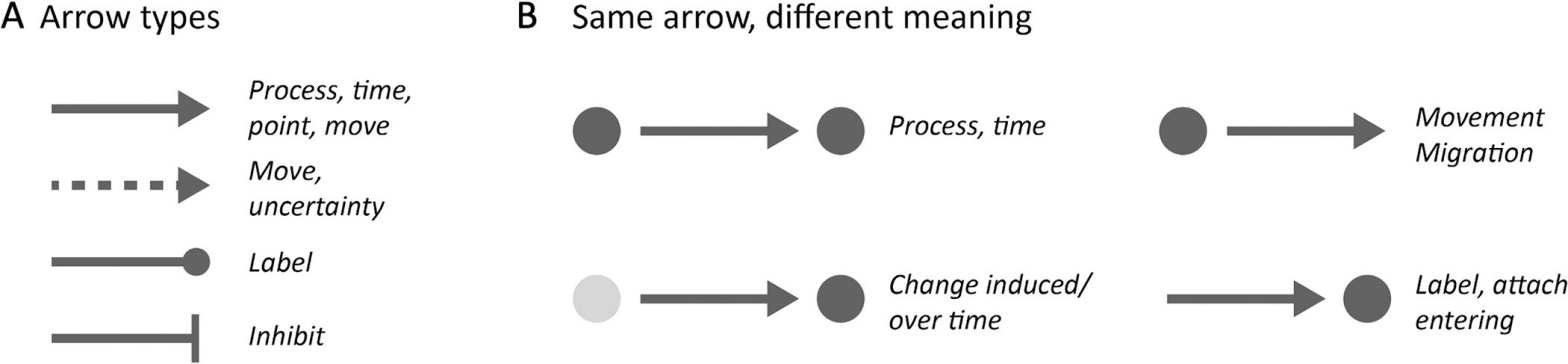
Common arrow types (A) and arrows in context (B).

In graphical abstracts, arrows have several distinct appearances and also distinct functions. Arrows also include arrowheads, lines with rounded tips or other end-marks (Fig 6A), and lines without any marks [17]. Remarkably, a single arrow type may convey distinct semantic meanings: an upward arrow may signify an upward movement, an increase, or a positive connotation, while a circular arrow can symbolize various temporal scales, from a day, to year, or an entire life cycle [17]. In many academic domains, arrows have also specialized applications, such as a corner/bent arrow that in molecular genetics illustrates transcription start sites [16]. Arrows can also depict various movements, representing phenomena like the passage of a molecule through a membrane, the migration of cells within a tissue, or the collective herd movement of animals. And finally, arrows and lines are also commonly used for labeling and directing attention to specific structures or regions of interest.

It is crucial that you clearly communicate the purpose of your arrows to your audience. When combining 2 different arrow types in a single graphical abstract, you should ensure they are visually distinct and explained. Moreover, the context in which an arrow is presented has substantial influence on how it’s perceived (Fig 6B).

Even with a clear layout and arrows, graphical abstracts can appear overwhelming. This feeling is rooted in the limitations of our visual system. Miller postulated the “Magical number 7,” suggesting that human sensory perception can effectively process only about 7 elements (plus or minus 2) at a time [18]. Of course, graphical abstracts typically comprise more than 7 elements. To address this challenge, design principles, often referred to as “Gestalt principles,” come into play, aiding in the organization of elements into interconnected units, or “chunks,” which enhances the information conveyed and reduces cognitive load [19].

Some of the design principles are especially helpful for graphical abstract design. “Proximity” suggests that elements can be grouped by minimizing their physical distance on the page. “Similarity” describes that elements form groups when they share common visual attributes. Such visual attributes, e.g., a shared color, pattern, or shape [20], may even lead to grouping when elements are not in close physical proximity. Grouping by similar appearance is helpful, e.g., in scatterplots, but can cause confusion if applied erroneously to non-grouped elements (see #8). “Closure” stipulates that elements within the same boundary are grouped, which explains the frequent use of boxes in design templates. However, it’s worth noting that boxes can often be replaced with white space to achieve a similar effect. The principle of “continuity” asserts that elements arranged along an invisible axis visually form a group, an idea that inspired Tufte to experiment with omitting x-axes in bar plots altogether [21]. And last, “similarity” suggests that elements arranged symmetrically appear grouped. These design principles are helpful for graphical abstracts but also valuable for improving your further designs such as scientific figures, as exemplified by Bang Wong [22,23].

## Rule 7: Text

The most effective way to ensure audiences comprehend complex insights with graphical abstracts is by seamlessly integrating text and visuals [24,25]. To captivate your audience, consider incorporating well-known keywords and phrases [7]. Text can also serve as a substitution when suitable images or pictograms are unavailable, particularly for specialized names or terminology, e.g., “acetylcholine.” Text is also important for labeling ambiguous or unusual visuals, icons, or arrows. For example, a circle you use could represent a molecule, an area, or a cell. While text offers additional clarifications, be sure to keep your titles and annotations concise, devoid of jargon, and limited to common abbreviations, all of which in general enhance readability and citations of scientific articles in general [26]. Lastly, text can play a dual role as a legend when the annotation mirrors the encoding style of associated visual elements. You may color a key word in the title in the same hue as the associated data in the abstract (see Fig 3).

## Rule 8: Colors

**Fig 7:**
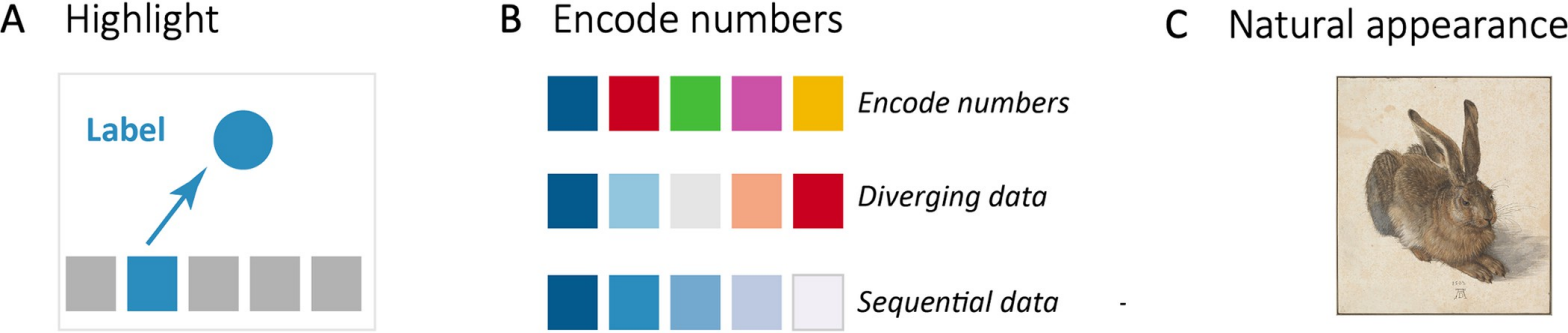
Color can highlight (A), encode numbers (B), or show natural appearance (C) in graphical abstracts. Be careful with your color choice when using a colored background. Image: Albrecht Dürer, Public domain, via Wikimedia Commons (https://commons.wikimedia.org/wiki/File:Albrecht_D%C3%BCrer_-_Hare,_1502_-_Google_Art_Project.jpg).

A key function of appealing colors in graphical abstracts is to engage your audience. Beyond that colors have further roles, color highlights, contrasts, encodes quantities, or represents the natural appearance of the depicted objects (Fig 7A–7C). When colors encode quantitative information, sequential or continuous data should be encoded with varying saturations of a single color, diverging data with e.g., two-color scheme, and for qualitative data you may vary the hue [27,28].

Several tools are available that may be helpful when selecting your color schemes. Colorbrewer by Cynthia Brewer (https://colorbrewer2.org/) is useful for choosing colors to encode numerical data, while Paletton (https://paletton.com/) enables the selection of attractive color combinations using a color wheel. These tools can assist in achieving harmonious appearances through adjacent colors or creating striking contrasts by employing complementary colors.

Consistency in color usage is important (see #6, principle of similarity). It is vital that you maintain the same color code and scheme within the abstract, and between the abstract and the main manuscript. A change in color is not merely a shift in aesthetics, it signifies a change in meaning. Colors, being instantly perceptible, should be used sparingly to prevent overwhelming the audiences and diverting their attention from the primary message. Hence, make your color choices with utmost care.

When selecting colors, you should ensure that they are accessible to your color blind audiences [29]. But more generally, you should consider possible limitations to visually impaired audiences. A comprehensive study provides an overview of accessibility in visualizations for different target groups (i.e., color-blind, visually impaired, and blind individuals) and various visual tasks [30]. A few steps help to improve accessibility: all figures, including graphical abstracts, must always be described with accompanying text. You may also be able to provide Alt-text descriptions for screen reader software. Additionally, also visually able audiences differ in their perception of color and contrast and therefore color should be avoided as the sole channel for key information (see also #7, labeling visuals). Beyond avoiding certain color combinations, like red-green for individuals with Deuteranopia, also low-contrast color combinations and many background colors may reduce visibility and thus accessibility. You can use numerous web-based tools (e.g., https://www.color-blindness.com/coblis-color-blindness-simulator/) or render your monitor display settings to assess legibility. WebAIM suggests a minimum contrast ratio of 4.5 to 1 for foreground and background colors and provides a tool for assessing color combinations (https://webaim.org/resources/contrastchecker/). Finally, maintaining a sufficiently high resolution is vital for ensuring accessibility, allowing your audiences to print or zoom in to your visualizations as needed.

## Rule 9: Tools for graphical abstracts

Graphical abstracts are typically prepared with the same software as posters and figures. Suitable are commercial (e.g., Adobe Illustrator, CorelDraw, Affinity Designer) or open-source (e.g., Inkscape) vector-design software. Vector-based graphics programs are particularly useful as they allow for zooming in and out of visualizations without quality loss. For most graphical abstracts PowerPoint will also produce sufficient results, especially when the canvas size is adjusted and slides are exported as vector graphics such as PDF. When saving your graphical abstract make sure that your images are not compressed to prevent pixelation artifacts.

A comprehensive article reviews many common software used for illustrations as well as their advantages, disadvantages and pricing is available [31]. If you wish to use the free vector graphic software Inkscape, you may consult a practical guide for biologists [32]. Inkscape is rapidly developing and now allows direct import of icons from icon libraries, as well as processing of images and data with scripts inside the software. The proprietary alternative to Inkscape is Adobe Illustrator, which is widely adopted by scientists and for which tutorials are available [33]. Another commercial software is CorelDraw which can, like Inkscape, incorporate icons from many web-based icon libraries.

In recent years, several web-based drawing softwares have become available, such as Canva or Figma. BioRender is a proprietary web-based software powered by a large biomedical icon library, which is an attractive feature to its users; however, their appearance, shape, color, and detail cannot be changed. A drawback to many labs is also BioRender’s continuous adaptation of licenses, while an advantage is its interface with public databases, such as the Protein Data Bank. Another web-based tool is Mindthegraph, which also offers in addition design consulting. A summary of tools is available [7].

Pictograms and icons can be imported in all programs, including the web-based tools, as SVG or PNG (see #2) and Inkscape even allows the direct, web-based import from icon libraries such as Bioicons or Reactome.

## Rule 10: Before, during, after: Feedback

Visual design is a dynamic and iterative process. Consequently, graphical abstracts should undergo several rounds of assessment and adjustment to avoid common pitfalls such as unclear reading directions [2] and inconsistencies in elements and style within the visualization.

Feedback can be actively sought and integrated at various stages: during the formulating of your key message, the drafting of your prototype, or the final polishing phase. As a best practice, the book Storytelling With Data in fact recommends allocating dedicated time for discussing the visualizations in every meeting [34]. As in every design of a human–computer interaction, also for graphical abstracts you may seek expert feedback, e.g., from a scientists or designers that regularly prepare graphical abstracts, as well as user feedback, e.g., from scientists or students who may read your paper.

General feedback principles [35] also apply to visual work. This means that feedback should be specific, tangible, and task-oriented and those seeking feedback should be clear in their request. In graphical abstracts, the audience must decode the visual representations. You can get feedback by observing how an expert or user is interacting with your graphical abstract, or by asking for their opinions. Ask what they see at first glance to see if the visual weight aligns with the key message. Ask about clarity of the layout and reading direction, including the meaning of arrows, and the comprehensibility of visual elements and colors. Alli Torban from Tableau, a visual design company, imparts additional guidance on the intricacies of soliciting and receiving feedback for visual designs [36].

When designing graphical abstracts in a team, we usually exchange rapid drafts or sketches of the graphical abstract several times before a solid idea emerges (Fig 1) and is then prepared for publication [37]. In our experience, the process of preparing a graphical abstract also serves as a valuable exercise to assess whether our key message is succinct. It also aids writing teams and grant writers in aligning toward a shared vision or objective. The graphical abstract thus serves as a valuable tool for bridging communication or knowledge gaps in transdisciplinary teams such as consortia of clinicians, engineers, and biologists.

## Conclusion

While initially graphical abstracts may seem like extra work for little reward, we hope that our 10 rules encourage you to start creating understandable and gorgeous graphical abstracts. A useful resource for educators wishing to teach graphical abstract preparation in a classroom setting is available from Agrawal and Ulrich, who provide templates for exercises and downloadable sample materials [9]. A quick guide, along with a PowerPoint template, is also available from Elsevier [38]. And for inspiration the British Medical Journal hosts a collection of infographics (https://www.bmj.com/infographics). Once you become familiar with the format of graphical abstracts, you may also wish to experiment with styles and forms. Usually, journals do not limit their authors: we have seen artistic, comic-style [39], and even hand-drawn (Fabio di Belvis: https://www.sciencedirect.com/science/article/pii/S0378517319307975?via%3Dihub) graphical abstracts.
